# Analyzing the Correlation between the Level of Serum Markers and Ischemic Cerebral Vascular Disease by Multiple Parameters

**DOI:** 10.1155/2015/972851

**Published:** 2015-11-01

**Authors:** Laibin Dong, Rongzhi Hou, Yuxia Xu, Jiaying Yuan, Lijun Li, Chao Zheng, Hehua Zhao

**Affiliations:** ^1^Department of Neurology, The Ninth People's Hospital of Zhengzhou, Zhengzhou 450000, China; ^2^Department of Ultrasound Diagnosis, Hospital of Henan Military Region, Zhengzhou 450000, China

## Abstract

*Objective.* To explore the serum markers associated with ischemic cerebral vascular disease (ICVD) and discuss their diagnostic value.* Methods.* Two hundred and eighty-eight patients with ICVD and one hundred and eighty healthy persons were enrolled as the case group and the control group, respectively. This paper then carried out the univariate and multivariate logistic regression analyses of their respective levels of serum markers, made combined analysis of related factors, and detected the diagnostic value.* Results.* Meta-analysis results showed that for ICVD patients the levels of CRP, S-100, TNF-*α*, HCY, NSE, and IL-6 were higher than those of the healthy persons, while the level of HDL was obviously lower than that of the healthy persons. The multivariate regression analysis indicated that the association between the level of HDL and TNF-*α* and the occurrence of ICVD was statistically significant (*P* < 0.05). The area under the curves (AUC) of receiver operating characteristic (ROC) curve of HDL and TNF-*α* was 0.916, with sensitivity of 90.91% and specificity of 76.47%.* Conclusion.* HDL has negative correlation with the occurrence of ICVD, while TNF-*α* was positively correlated with it. The combination test of HDL and TNF-*α* could raise the accuracy of ICVD diagnosis.

## 1. Introduction

In recent decades, along with the rapid economic development and people's living standards improvement, ischemic cerebral vascular disease (ICVD) has become an important factor threatening human health all over the world. Currently, the mortality of ICVD, second ranked, has been one of the main causes of adult disability. ICVD mainly includes transient ischemic attack (TIA) and cerebral infarction (CI). Transient ischemic attack is a transient blood supply shortage occurring in carotid or vertebral-basilar artery system, causing focal cerebral ischemia and leading to sudden, transient, and reversible neurological dysfunction. Its typical clinical symptoms last no more than one hour and have no radiographic evidence of acute cerebral infarction. If TIA was not under the control in time it may lead to the onset of cerebrovascular disease eventually. The incidence of cerebral infarction is next only to TIA. Due to cerebral vascular severe stenosis or occlusion and because collateral circulation is insufficient to maintain the requirement of brain metabolism, the ischemic necrosis of the brain tissue occurs leading to cerebral infarction. Most ICVD patients have no premonitory symptoms, and considering its features such as high incidence, disability, mortality, and recurrence rate, the early diagnosis of ICVD is particularly important.

Meta-analysis is a quantified overview of the literature. Taking a number of results of independent studies for the same issue as the research object, meta-analysis conducts systematic and objective quantitative comprehensive analysis of various research results by using appropriate statistical methods. Meta-analysis is a series of processes in which the results of multiple studies are summarized and the combined effect sizes are analyzed and evaluated.

Based on literature search, using meta-analytical method, this study conducted a comprehensive analysis of the case-control study literatures related to serum markers of ICVD domestically and abroad, and at the same time the further detection of the level of the relevant markers was run, as well as the analysis of their relations between ICVD and their diagnostic value, to offer a reference basis for early diagnosis and screening of ICVD.

## 2. Material and Methods

### 2.1. Meta-Analysis

#### 2.1.1. Data Sources

Clinical research literatures published at home and abroad about the ICVD serum markers from January 1, 2000, to August 1, 2014, were enrolled in this study. Literature sources include Wanfang Data, CNKI, VIP Journal Integration Platform (VJIP), PubMed, and other Chinese and English databases. The corresponding Chinese keywords' meanings are “cerebrovascular disease”, “ischemic”, and “serum marker”. The English keywords are “cerebral vascular disease”, “ischemic”, and “serum marker”. The full text of the included literatures is either Chinese or English.

#### 2.1.2. Data Selection Criteria

*Inclusion Criteria.* The study subjects were diagnosed as ICVD in accordance with the recognized standards. As for the researches conducted by the same institution or person on the same subjects but published in different journals, we selected their most recent and complete documentation. Research factors of literatures were about serum markers of ICVD. Experimental design was case-control study. Articles publication time was also included.

*Exclusion Criteria.* The following were excluded: repeat papers, literature reviews, comments or lectures, literatures without statistical contents, and literatures without (correct) data analysis methods.

#### 2.1.3. Statistical Analysis

The included literatures were performed meta-analysis by software RevMan5.1. The research data were conducted heterogeneity test and their combined SMD values and 95% confidence intervals (CI) were calculated. When the heterogeneity test results *P* were greater than 0.05, the fixed effects model was adopted. When *P* was less than 0.05, the random effects model was used. Then the combined values of SMD and 95% CI were output, and *Z*-test on combined statistics was conducted. *P* ≥ 0.05 indicated that multiple combined statistics had no statistical significance, while *P* < 0.05 makes that statistically significant.

### 2.2. Statistical Analysis of Serological Markers

#### 2.2.1. General Information

In this study, 288 cases of ICVD patients from our hospital during October 2009 to October 2014 were enrolled as the subjects, with 180 males and 108 females, aged from 17 to 89 years with an average age of 59.3 ± 9.4 years. All patients received brain computed tomography (CT), carotid color ultrasonography, electrocardiograph (ECG), magnetic resonance imaging (MRI), and angiography test to rule out other causes of brain damage. One hundred and eighty healthy examination persons who matched the patients group in age and sex were selected as the control group, with 108 males and 72 females, ranging in age from 40 to 82 years with an average age of 64.7 ± 11.2 years.

#### 2.2.2. Methods

Important indicators of the patients group during hospitalization were collected, as well as that of control group, including homocysteine (HCY), C-reactive protein (CRP), high density lipoprotein (HDL), S-100, tumor necrosis factor *α* (TNF-*α*), neuron-specific enolase (NSE), interleukin 6 (IL-6), and other indicators as independent variables and whether the patient is sick or not as the dependent variable. Software SPSS19.0 was utilized for dichotomy univariate and multivariate logistic regression analysis of the data. When *P* < 0.05, that was considered statistically significant, and the serum markers related to ischemia cerebrovascular disease were screened.

The combined detection sensitivity and specificity of serum markers screened by logistic regression were calculated. Software SPSS19.0 was adopted to draw the ROC curve and calculate the AUC of the curve.

## 3. Results

### 3.1. Meta-Analysis Results

#### 3.1.1. Literature Search Results

According to the inclusion and exclusion criteria, this study ultimately selected 48 relevant literatures for statistical analysis, containing 31 Chinese literatures and 17 English literatures.

#### 3.1.2. Literature Heterogeneity Test Results

Heterogeneity test was conducted to the results of seven serum markers CRP, S-100, TNF-*α*, HCY, NSE, HDL, and IL-6 of ICVD. The results were shown in Table 1. It was found that the results of all the serum markers were inconsistent. *P* values were always less than 0.00001; thus the random effects model was used.

#### 3.1.3. Meta-Analysis Results of Each Serum Marker

Seven kinds of serum markers indicators CRP, S-100, TNF-*α*, HCY, NSE, HDL, and IL-6 were analyzed by meta-analysis, and the results were in Table 2. It showed that the SMD combined effect and the bound of 95% CI of CRP, S-100, TNF-*α*, HCY, NSE, and IL-6 were all more than 0. Additionally, the transverse line of 95% CI was on the right side of the void vertical line, and the *P* values of the combined test were all less than 0.05, and the difference was statistically significant; thus the levels of the six serum markers CRP, S-100, TNF-*α*, HCY, NSE, and IL-6 of ICVD patients were higher than those of the healthy ones. In contrast, the SMD combined effect size and the bound of 95% CI of HDL were all less than 0. In addition, the transverse line of 95% CI was on the left of the invalid vertical line, and the *P* values of the combined test were all less than 0.05, and the difference was statistically significant. Hence, it was concluded that the concentration of HDL in ICVD patients was lower than that of the normal people. In summary, these seven serum indicators CRP, S-100, TNF-*α*, HCY, NSE, HDL, and IL-6 were all associated with ICVD.

### 3.2. Logistic Regression Analysis of Serum Markers

Regarding HCY, CRP, HDL, S-100, TNF-*α*, IL-6, and NSE as independent variables and illness or no illness as the dependent variable, this paper conducted a dichotomy logistic regression analysis using SPSS19.0. The results of univariate regression analysis showed the association between CRP, HDL, TNF-*α*, IL-6, and NSE and the occurrence of ICVD was statistically significant (*P* < 0.05, Table 3). After multivariate regression analysis of all independent variables, it was found that only the association between HDL and TNF-*α* and the occurrence of ICVD was statistically significant (*P* < 0.05, Table 4).

### 3.3. The Value of HDL and TNF-*α* in ICVD Diagnosis

**Figure 1:**
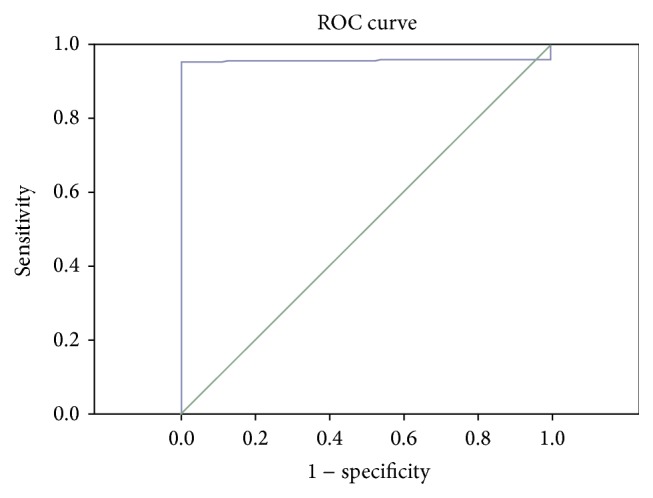
ROC curve of HDL and TNF-*α* combined detection of ischemic cerebrovascular disease.

The ROC curve of HDL and TNF-*α* combined detection of ICVD is shown in Figure 1. The AUC was 0.916 and 95% CI was 0.889~0.943. The sensitivity of HDL and TNF-*α* combined detection was 90.91% and the specificity was 76.47%.

## 4. Discussion

ICVD is a cascade process which was induced by ischemic cytokines, no matter the patient is infected or not, there are enduring inflammatory reactions at its early stage, and the atherosclerosis is the main etiology and risk factor. Atherosclerosis is a chronic inflammatory process, in which the vascular wall cells are stimulated by harmful substances and the cytokines are produced. The inflammatory reaction involving multiple cytokines plays a key role in the formation of ICVD [1–3].

Tumor necrosis factor TNF-*α* not only has a cytotoxicity and the growth inhibition on tumor cells, but also can mediate the production of IL-1, IL-6, and other inflammatory mediators to participate in the collective physiopathologic process. Studies have shown that TNF-*α* may affect the activity of vascular endothelial cells and regulate the vascular inflammation process [4].

S-100 protein is an EF-hand calcium-binding protein, which is widely distributed in various tissues and has a wide range of biological activities [5].

C-reactive protein, induced by cytokines such as IL-6 and TNF-*α*, a common acute phase inflammatory reactive protein in human serum, can only be synthesized by the stem cells. C-reactive protein has been taken as an inflammatory marker since it usually has significant increase under such conditions like severe bacterial infections, atherosclerosis, vascular injury, ischemia, and necrosis. An increasing number of studies have suggested there are close relationships between CRP and atherosclerosis as well as cerebral infarction [6]. Studies reported that CRP is an independent predictor of acute ischemic stroke. Through the activation of the classical complement pathway, cytokine production, and complement-related inflammatory response, CRP will accelerate the acute inflammatory injury, which may also promote the formation of atherosclerosis thrombus and the occurrence of complications [7].

Studies have shown that the elevation of HCY level is a risk factor of cerebrovascular disease and can result in arterial smooth muscle proliferation and peroxidation by causing atherosclerosis [8–10].

Neuron-specific enolase (NSE) is a ubiquitous key glycolytic pathway enzyme in vivo. At present, researches indicate that the NSE levels of stroke patients are significantly higher than that of the normal and the elevated levels are related to the infarct volume of brain tissue; therefore, NSE can reflect the severity of patients indirectly [11–13].

The present study collected the relevant literatures since 2000 and chose literatures with a relatively high credibility. After the meta-analysis of ICVD serum markers, reported in the literature [14], it was found that the related literature heterogeneities of the seven serum markers CRP, S-100, TNF-*α*, HCY, NSE, HDL, and IL-6 were relatively high. A random effects model was then employed to analyze and compare various serum markers in the experimental and control groups. Those in the experimental groups were all effective for ICVD, and the combined test *P* value was less than 0.05, while the OR values and 95% CI of CRP, S-100, TNF-*α*, HCY, NSE, and IL-6 were all greater than 0, indicating that six serum indicators CRP, S-100, TNF-*α*, HCY, NSE, and IL-6 of patients with ICVD were higher than those of the normal, and their increasing levels were the unfavorable factors of ICVD. The OR value and 95% CI of HDL were less than 0, which suggested that the HDL concentration of patients with ICVD was lower than that of the normal and its increasing level was the protective factor for ICVD.

Through the regression analysis of the association between CRP, S-100, TNF-*α*, HCY, NSE, IL-6, and ICVD, the univariate analysis showed that the association between CRP, HDL, TNF-*α*, IL-6, and NSE and ICVD was statistically significant (*P* < 0.05). The multivariate analysis showed association between HDL and TNF-*α* and ICVD was statistically significant (*P* < 0.05); besides, HDL was negatively correlated with the occurrence of ICVD, whereas TNF-*α* was positively correlated with ICVD. HDL mainly involved the process of reverse transport of cholesterol and the inhibition of the uptake of vascular smooth muscle to HDL and platelet aggregation, restores vascular endothelial cells function, and eliminates atherosclerotic plaque. Numerous epidemiological studies tend to believe that elevating the HDL levels can dramatically reduce the incidence of ICVD, and the low HDL levels are an independent risk factor for ICVD [15], which are consistent with the results of this study. Hao et al. [16] believed that the HDL levels were related to ICVD state; that is, the greater the lesion area was, the more distinctly the HDL levels decreased, so there was a negative correlation between them. Studies of Lindenstrøm et al. [17] suggested that the lower the HDL level is, the higher the incidence of ICVD is, and they were negatively correlated with each other, which is also consistent with the results of this study. Studies have indicated that TNF-*α* not only is related to the inflammations after cerebral ischemia, but also plays a role in the formation of thrombosis. It can also act on vascular endothelial cells, and all of these effects have certain relationships with ICVD; meanwhile, the TNF-*α* level in acute ICVD patients' blood has a significant increase [18, 19].

The HDL and TNF-*α* combined detection is the crucial hint to the diagnosis and detection of ICVD, and the AUC of its ROC curve was 0.916, indicating that the diagnosis effect was preferable. Sensitivity of the combined detection was 90.91%, specificity 76.47%, suggesting that the combined analysis of HDL and TNF-*α* can be taken as a reminder for doctor whether the patients were under high risk of ICVD.

## 5. Conclusions

The combined detection of HDL and TNF-*α* is significant for the diagnosis of ICVD, and it is also a vital hint for the occurrence of ICVD; at the same time, it can raise the diagnosis accuracy, which is clinically significant for patients' in-time treatment and conditions judgment. Their levels' dynamic monitoring can be considered as a reference for the evaluation of ICVD's development and recovery process, dramatically decreasing the cost of diagnosis and recovery, and, particularly, it is promising in application at primary hospitals.

## Figures and Tables

**Table 1:** Heterogeneity test results.

Serological markers	Literature number	Experimental group	Control group	Heterogeneity test	
				*χ* ^2^	*P*
CRP	13	1460	5502	3596.58	<0.00001
S-100	8	608	261	155.93	<0.00001
TNF-*α*	9	464	387	343.27	<0.00001
HCY	7	862	550	883.52	<0.00001
NSE	8	645	386	380	<0.00001
HDL	11	1554	5855	208.63	<0.00001
IL-6	9	396	369	436.73	<0.00001

**Table 2:** Meta-analysis results.

Serological markers	Literature number	Experimental group	Control group	SMD	95% CI	Combined effect size test	
						*Z*	*P*
CRP	13	1460	5502	5.17	3.02~7.32	4.71	<0.00001
S-100	8	608	261	1.06	0.89~1.23	12.43	<0.00001
TNF-*α*	9	464	387	2.18	1.99~2.38	21.84	<0.00001
HCY	7	862	550	12.70	11.90~13.50	31.09	<0.00001
NSE	8	645	386	1.93	0.66~3.20	2.97	0.003
HDL	11	1554	5855	−0.35	−0.41~−0.28	10.25	<0.00001
IL-6	9	396	369	9.47	8.35~10.60	16.55	<0.00001

**Table 3:** Univariate analysis of the risk factors of ischemic cerebrovascular disease.

Serum markers	*B*	Wald	*P*	OR (95% CI)
HCY	0.026	1.610	0.205	1.026 (0.986~1.069)
CRP	0.077	3.886	0.049	1.081 (1.0~1.167)
HDL	−0.134	5.873	0.015	0.874 (0.784~0.975)
S-100	0.546	1.613	0.204	1.780 (1.150~2.845)
TNF-*α*	0.682	7.248	0.007	1.886 (1.208~2.931)
IL-6	0.012	4.229	0.040	1.018 (0.987~1.052)
NSE	0.076	5.031	0.025	1.078 (1.017~1.174)

**Table 4:** Multivariate analysis of the risk factors of ischemic cerebrovascular disease.

Serum markers	*B*	Wald	*P*	OR (95% CI)
HCY	0.025	1.260	0.262	1.025 (0.982~1.070)
CRP	0.066	2.540	0.111	1.068 (0.985~1.158)
HDL	−0.127	4.836	0.028	0.881 (0.787~0.986)
S-100	0.396	0.758	0.384	1.543 (1.076~2.041)
TNF-*α*	0.668	6.788	0.009	1.813 (1.194~2.748)
IL-6	0.010	2.598	0.107	1.012 (0.978~1.050)
NSE	0.065	3.469	0.063	1.066 (1.005~1.136)
